# Vaccine Therapy of Typhoid

**Published:** 1914-08

**Authors:** 


**Vaccine Therapy of Typhoid.** E. Allenbach, Muench. Med. Woch., page 978, 1914, treated 8 cases, 3 had relapses, 2 haemorrhages from the bowel, one died. It neither shortened the fever nor improved the general condition.

